# 纳升解吸电喷雾电离质谱成像方法的近期研究进展

**DOI:** 10.3724/SP.J.1123.2024.07013

**Published:** 2025-01-08

**Authors:** Jingbo WANG, Xiaolan LI, Ruixia FAN, Ping LÜ, Ruichuan YIN

**Affiliations:** 四川大学华西药学院, 四川 成都 610041; West China School of Pharmacy, Sichuan University, Chengdu 610041, China

**Keywords:** 纳升解吸电喷雾电离, 常压质谱成像, 脂质结构分析, 空间蛋白质组, nanospray desorption electrospray ionization, ambient mass spectrometry imaging, structural characterization of lipid, spatial proteomics

## Abstract

常压质谱成像无需复杂的样品处理步骤,能在大气压下直接对组织切片中成百上千种分析物进行原位分析。目前,该领域正处于蓬勃发展的时期。已报道的常压电离技术种类众多,并呈现出“百花齐放”的良好态势;其中,纳升解吸电喷雾电离(nano-DESI)是近年发展迅速的一种基于液滴萃取的常压电离技术。脂质结构的复杂性给nano-DESI脂质成像带来了极大的挑战,而新型nano-DESI联用技术使脂质精细结构的鉴定与成像成为可能。与电喷雾电离(ESI)类似,nano-DESI倾向于产生多电荷态的分子离子,因而在蛋白质大分子的成像中具有显著的优势。本文聚焦于近3年nano-DESI在离子源研发、脂质结构分析和空间蛋白质组学方面的研究进展,并探讨了nano-DESI领域中亟需解决的关键问题。

## Abstract (en)

Ambient mass spectrometry imaging (MSI) enables hundreds of analytes in tissue sections to be directly mapped at atmospheric pressure with minimal sample preparation. This field is currently experiencing rapid growth, with numerous reported ambient ionization techniques resulting in a “hundred flowers bloom” situation. Nanospray desorption electrospray ionization (nano-DESI), developed by the Laskin group in 2010, is a widely used liquid-extraction-based ambient ionization technique that was first used for mass spectrometry imaging of tissue in 2012. The nano-DESI probe comprises a primary capillary and a nanospray capillary, with the latter efficiently transferring analyte-containing droplets via a tiny liquid bridge formed between the probe and sample surface, thereby enabling nanoelectrospray ionization (nano-ESI) in front of the inlet of a mass spectrometer. The advantages of nano-DESI MSI include minimal sample preparation, high spatial resolution, and high sensitivity. These features are well-suited for imaging various sample types, including frozen tissue sections, microbial communities, and environmental samples. A PubMed-database search using the “nano-DESI” keyword revealed 72 related articles in the 2010-2024 period, with 34 of them published between 2021 and 2024, which indicates that nano-DESI has rapidly developed as an ambient ionization technique over recent years. Herein, we briefly introduce key nano-DESI-MSI research progress reported in the past three years with the aim of better understanding and facilitating the use of this technology. We first discuss advances in ion-source development. Since no commercial nano-DESI source exists, designing and constructing ion sources remain technical challenges that limit its development. Nano-DESI has been successfully coupled with various types of mass spectrometer, including LTQ Orbitrap, quadrupole-Orbitrap (Q Exactive), 6560 IM QTOF, timsTOF Pro2, triple quadrupole, and FTICR. These couplings have significantly expanded the applications range of the nano-DESI technique. Secondly, lipid analysis is a major nano-DESI-MSI applications area. While the complexities of lipid structures present great challenges for nano-DESI MSI, new nano-DESI coupling techniques have enabled the identification and imaging of fine lipid structures. Several novel imaging methods have recently been introduced to address difficulties associated with identifying lipid structures, such as distinguishing carbon-carbon double bonds (C=C) and *sn*-positional isomers. We finally highlight recent research progress in the nano-DESI MSI of intact protein assembles and proteoforms, which is a growing hotspot in the field. Unlike small lipid molecules, large protein molecules are very challenging to image and consequently demand higher instrumental performance (e.g., ionization efficiency, mass range, and sensitivity). In a similar manner to the ESI technique, nano-DESI tends to generate multiply charged molecular ions, which endows it with a significant advantage when imaging large protein molecules. Recent years have witnessed important nano-DESI-MSI progress for studying protein-ligand interactions and identifying and imaging endogenous proteoforms. In summary, this article focuses on nano-DESI research progress in terms of ion-source development, lipid-structure analysis, and spatial proteomics over the past three years and discusses key challenges that need to be addressed in the field.

纳升解吸电喷雾电离(nanospray desorption electrospray ionization, nano-DESI)是Laskin团队^[1,2]^于2010年开发的一种基于液滴萃取的常压离子化技术,2012年首次被用于组织质谱成像(mass spectrometry imaging, MSI)。经过十余年的迭代更新,nano-DESI MSI已成为一种认可度较高的、具有独特辨认度的成像方法。与经典的DESI技术类似,nano-DESI样品处理步骤简便,只需制备冷冻组织切片即可,并不需要样品清洗和涂敷基质,能在天然环境下进行样品的原位分析。与经典的DESI技术不同的是,nano-DESI中溶剂流速(≤ 500 nL/min)远低于DESI流速(3~15 μL/min),通常不需要雾化气体来辅助脱溶剂;nano-DESI探针由初级毛细管和纳喷毛细管构成,通过液滴接触样品表面,不仅可获得微米级的小液滴,而且不会破坏样品表面;纳喷毛细管能高效地转移含有分析物的液滴,使其在质谱入口发生纳升电喷雾电离(nano-ESI),因而nano-DESI灵敏度与nano-ESI技术相当(图1)^[1]^。简而言之,nano-DESI将微液滴萃取和nano-ESI融合在一起,具有高空间分辨率和高灵敏度等优点,适用于不同类型样品的成像分析,包括冷冻组织切片、微生物群落和环境样本等^[3]^。

**图1:**
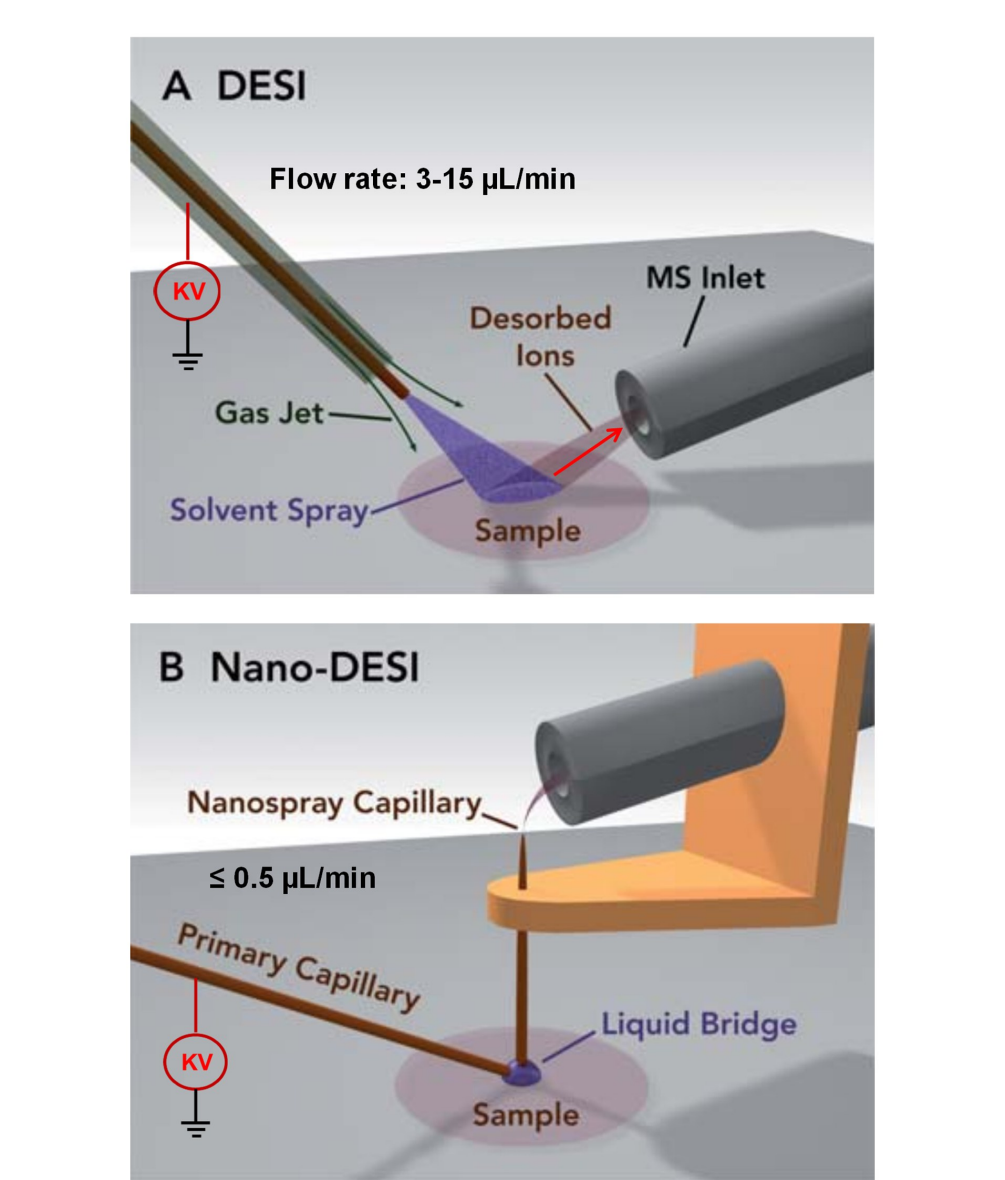
(A) DESI和(B)nano-DESI的示意图^[1]^

如图2所示,nano-DESI MSI实验的一般流程包括:1)nano-DESI探针的精细加工,包括拉伸、研磨以及测量尖端直径等;2)nano-DESI探针的精确组装,包括优化初级毛细管与纳喷毛细管之间的相对位置,调整纳喷毛细管与质谱入口之间的距离等;3)成像数据采集;4)成像数据的可视化及定量分析^[3]^。Nano-DESI MSI的空间分辨率取决于探针与样品表面之间液滴(liquid bridge)的尺寸,而液滴尺寸与毛细管尖端直径、毛细管的相对位置和纳喷毛细管与质谱入口之间的距离等因素密切相关。因而,毛细管尖端大小应视所需的空间分辨率而定,对于单细胞分辨率(≤10 μm),初级毛细管与纳喷毛细管的尖端直径需要控制在10~25 μm范围内。此外,恒定距离成像是保证高质量质谱成像的一个重要因素。

**图2:**
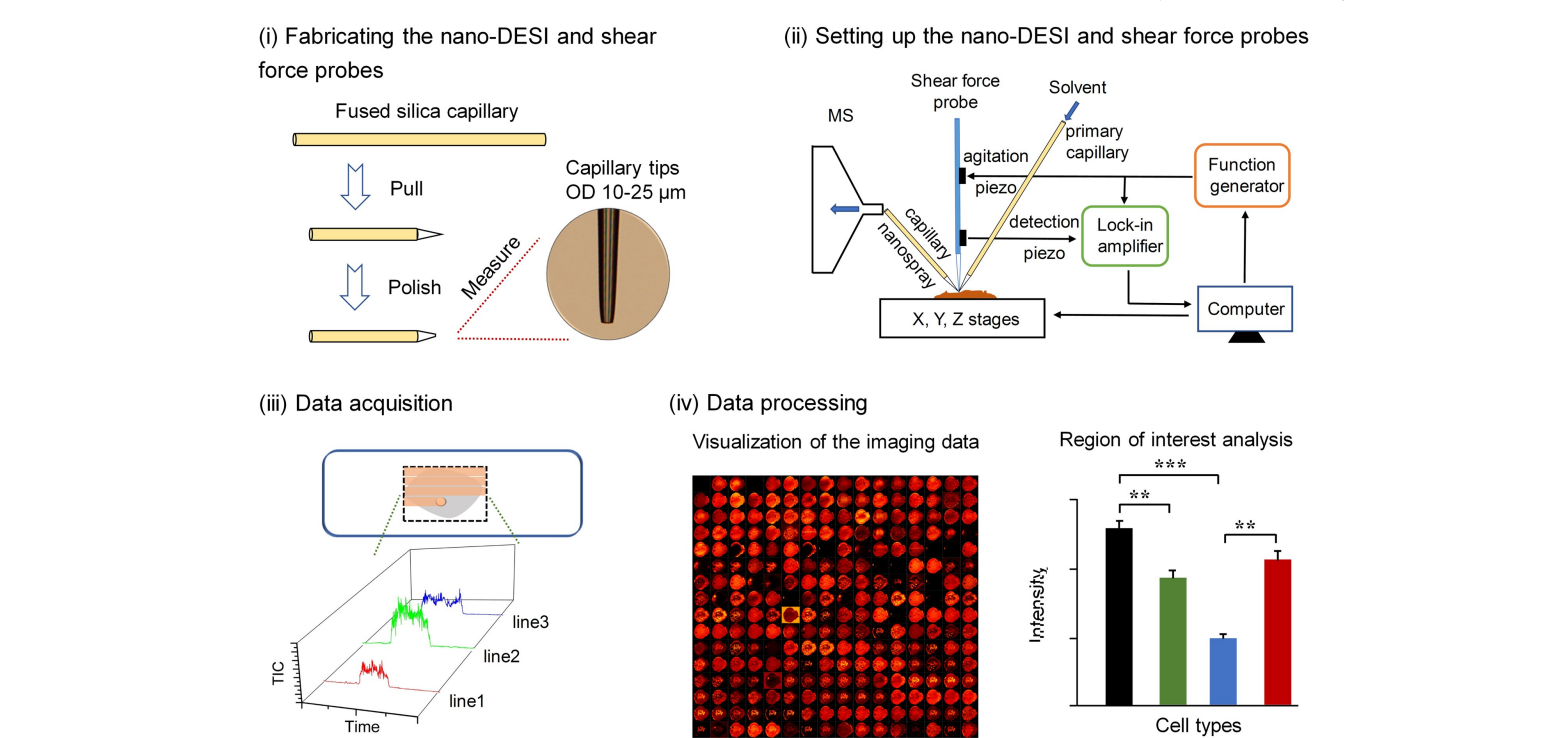
nano-DESI MSI的一般流程^[3]^

本团队^[3]^采用剪切力定位系统(剪切力显微镜),能够精确地控制nano-DESI探针与样品之间的距离。该系统使用尖锐的剪切力探针(约10 μm)来测量探针与样品表面的距离,并通过计算机的反馈回路实时调整距离,使其保持在一个恒定的数值。

从PubMed网站上,以nano-DESI为关键词进行搜索,结果显示了72篇相关论文(2010-2024年),其中34篇论文发表于2021-2024年,可见nano-DESI是近期快速发展的一种质谱成像方法。本文将简要介绍近3年nano-DESI MSI的重要研究进展,使更多的科研人员能够了解和使用这项技术。首先,介绍离子源研发方面的进展,离子源的设计与构建是nano-DESI MSI的技术难点,也是制约其发展的重要因素之一;其次,脂质成像一直是nano-DESI MSI的主要应用领域,近期一些新颖的成像方法被提出,用于解决脂质结构鉴定的难题,如碳-碳双键(C=C)和*sn*-位置异构体等;最后,重点介绍nano-DESI在空间蛋白质组学研究中的应用,这是近年来nano-DESI MSI领域的研究热点。

## 1 Nano-DESI离子源的开发

离子源是nano-DESI MSI平台的核心装置,由于没有商业化的nano-DESI源,科研人员需要根据质谱仪的结构自行设计与构建离子源。Laskin团队^[3⇓-5]^构建了一系列不同类型的nano-DESI源,分别用于线性离子阱-静电场轨道阱(LTQ Orbitrap)、四极杆-静电场轨道阱(Q Exactive)、离子淌度-四极杆-飞行时间(6560 IM QTOF)和捕集离子淌度-飞行时间(timsTOF Pro2)质谱仪等。他们基于LTQ Orbitrap质谱仪搭建了一款悬挂嵌入式nano-DESI源,这是一款经典的、应用较多的nano-DESI系统(图3A)。该系统包括三维平移台、nano-DESI探针、微型定位器、手持显微镜、微量注射泵和剪切力探针等。基于这一平台,他们通过精细加工nano-DESI探针和整合剪切力显微技术,实现了单细胞分辨率质谱成像(空间分辨率≤10 μm)^[3]^。

**图3:**
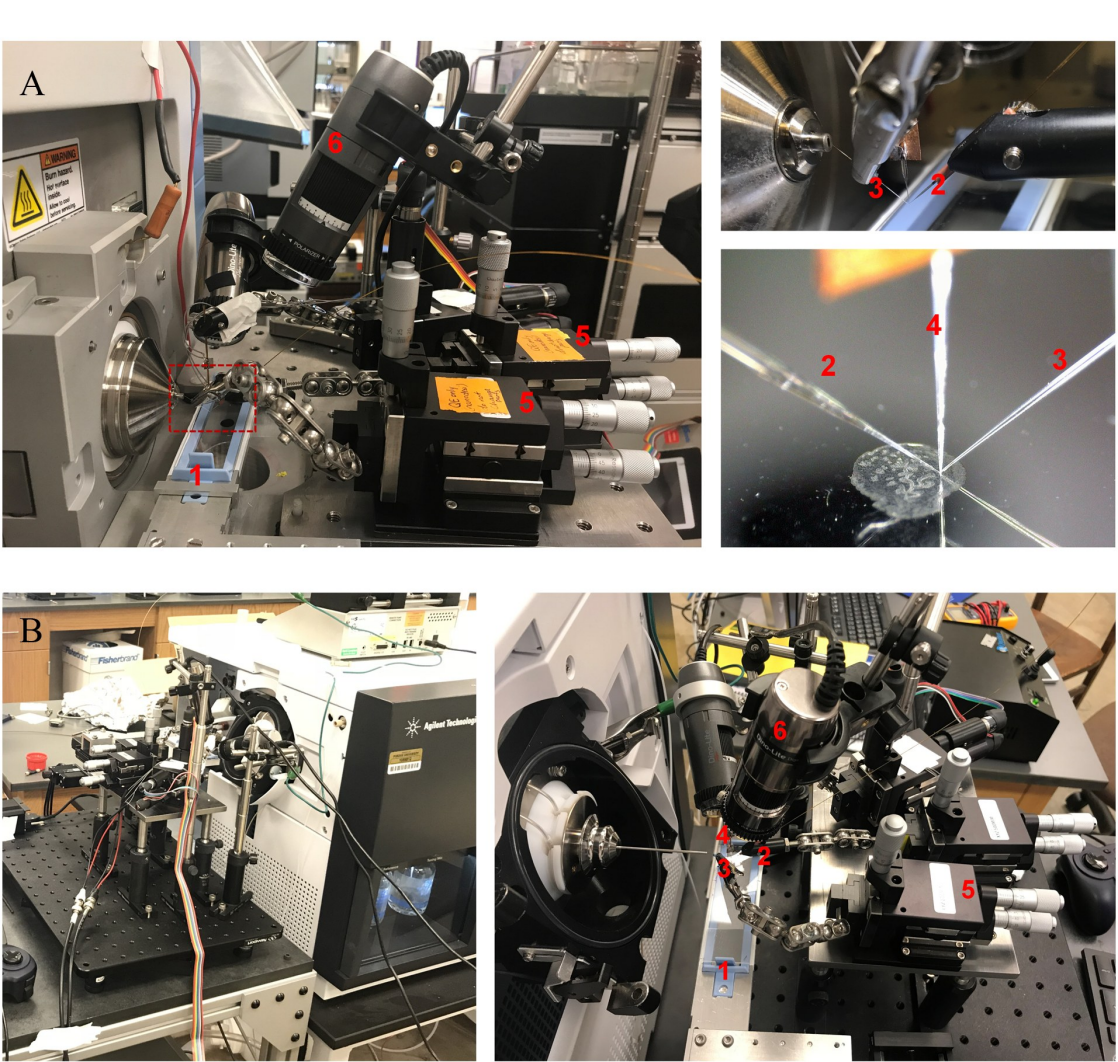
(A)基于LTQ Orbitrap质谱仪的悬挂嵌入式 nano-DESI源^[3]^和(B)通用型的独立式nano-DESI源^[4]^

在此基础上,该团队^[4,5]^研发了具有更好仪器兼容性的分离独立式离子源(图3B),并与离子淌度(IM)质谱联用,通过测量碰撞截面(CCS)提供分子结构信息,能够区分脂质的同分异构体(isomer)和同量异位素(isobar),并且揭示了它们在小鼠子宫和脑组织中的独特分布。比如磷脂酰胆碱(PC)32∶1的同分异构体分别具有不同的IM值(1.410和1.424), 1.410对应的异构体在小鼠脑组织海马区含量较高,而另一个在小脑区含量较低;[PC 34∶1+K]^+^和[PE O-40∶7+Na]^+^(PE为磷脂酰乙醇胺)的质量差仅为0.2 mDa,常规高分辨质谱仪很难区分这两个离子,而IM则能有效分离这对离子。IM MSI数据表明[PC 34∶1+K]^+^和[PE O-40∶7+Na]^+^分别富集在小鼠脑灰质和白质区域中。此外,Vandergrift等^[6]^首次将nano-DESI与傅里叶变换离子回旋共振质谱(21T FTICR MS)联用,这一联用融合了nano-DESI和21T FTICR的技术优点,同时具有高空间分辨率和高质量分辨率。通过使用该方法,他们展示了脂质同位素精细结构对应的离子图像(比如PE 40∶6和其^13^C同位素),还能区分质量相近离子(可分辨的最小质量差为8.96 mDa)的空间分布特征。

Nano-DESI探针的性能是决定成像质量的关键因素。传统的nano-DESI探针由两个独立毛细管构成,毛细管的精细加工和位置调整均需要耗费大量的时间和精力,而且毛细管尖端容易损坏。为了克服这些弊端,Laskin团队^[7]^开发了一种集成式微流探针(iMFP),巧妙地在石英玻片上构建两个微流通道,以代替初级毛细管和纳喷毛细管。这种探针具有“即插即用”和不易损坏等优点,在扫描速率高于传统探针10~15倍的条件下,几乎没有损失分子覆盖度和整体成像质量。重要的是,通过采用更小尺寸的通道和全新的采样口设计,iMFP能实现8~10 μm的空间分辨率,完全达到传统探针的最优性能^[8]^。该团队^[9]^进一步简化了iMFP的制备工艺,采用选择性激光辅助刻蚀(SLE)技术,取代了耗时耗力的传统光刻和键合工艺,并且优化了采样端口和电喷雾喷头的设计。这些措施显著提升了iMFP的制备效率,又能保持集成式探针的优异性能。

## 2 Nano-DESI脂质成像方法的新进展

结合碰撞诱导解离(CID)模式,常规nano-DESI MSI技术能用于脂质种类和支链组成的鉴定和成像分析,但难以直接识别脂质位置异构体。Lillja等^[10]^利用银离子衍生化与nano-DESI四级质谱(MS^4^)相结合的方法,在小鼠脑组织中成功鉴定了PC的*sn*-位置异构体。PC-银离子加合物通过迭代丢失头部基团和氢化银分子生成不稳定的碳正离子,进一步解离产生与*sn*-1或*sn*-2位酰基链对应的诊断离子。通过检测MS^4^特征离子,他们定量分析了小鼠脑皮质、胼胝体和海马下区中多种*sn*-位置异构体的含量,包括PC 16∶0_18∶1、18∶0_18∶1和20∶1_16∶0异构体等。Guo等^[11]^基于双线性离子阱质谱仪,设计了一个新型的气动辅助nano-DESI MSI平台。其创新之处如下:1)在纳喷毛细管末端带通入氮气辅助分析物的离子化,从而提升检测灵敏度;2)研制了一种新型的激光传感探头,用于实时调整nano-DESI探针与样品表面之间的距离;3)双线性离子阱能够采集多级质谱数据。他们将经典Paternò-Büchi(PB)反应和nano-DESI MS^3^结合,利用在紫外光照条件下PB试剂与C=C能发生[2+2]环化加成反应的特点,建立了一种同时鉴定和成像磷脂的脂肪酰链组成、*sn*-和C=C位置异构体的方法。使用该方法,他们揭示了多种PC和PE双键位置异构体在人肝脏组织的癌细胞区、纤维化区和正常组织中的差异化分布。例如,PC(16∶0 _18∶1比PC(18∶1 _16∶0)在肝癌区域中分布更丰富;PC(16∶0 _18∶1)的*n*-7、*n*-9和*n*-10双键位置异构体在人肝脏组织中也表现出不同的分布,其中*n*-7和*n*-9位置异构体在肝纤维化区域中丰度较低,*n*-10异构体在癌细胞区内丰度较高。

除了上述的多级质谱成像以外,近期其他数据采集模式(如选择离子监测(SIM)和多反应监测(MRM))与nano-DESI的联用也拓展了nano-DESI脂质成像方法的适用范围^[12,13]^。Laskin团队^[12]^将nano-DESI MSI与SIM相结合,通过设置一系列相邻的SIM窗口来同时获取多种分析物的高灵敏MSI数据。该方法显著提高了类二十烷酸(脂质信号因子)的检测灵敏度,实现了小鼠肾脏中多种低丰度类二十烷酸的成像分析;此外,该方法同样适用于生物和环境样品中其他低丰度分析物的测定。他们还将nano-DESI MSI平台与三重四极杆(QqQ)质谱仪联用,结合MRM模式,开发了能空间分辨同分异构体和同量异位素的一种简易方法^[13]^。MRM成像不仅具有高灵敏度的优点,特别适用于靶向分析低丰度分析物,而且可提供足够的结构信息,以区分质量相近或相同的离子。该方法不依赖高分辨率质谱仪,使用单位质量分辨率MRM模式就能区分磷脂同量异位素,而在全扫描模式下,需要超高质量分辨率(> 300万)才能分辨这些同量异位素。比如[PC 32∶1+K]^+^和[PE P-38∶6+Na]^+^质量差仅为0.2 mDa,在全扫描模式下需要385万分辨率才能区分这对离子,而通过检测母离子和特征子离子能够有效地分离它们。这些数据采集模式极大地增强了nano-DESI MSI的灵敏度、选择性和特异性。

## 3 基于nano-DESI的空间蛋白质组学研究

与脂质小分子不同,蛋白质大分子的质谱成像是一项极具挑战性的工作,对仪器性能(离子化效率、质量范围、灵敏度等)提出了更高的要求。近年来,nano-DESI MSI在空间蛋白质组学研究中具有十分优异的表现。Cooper团队^[14]^采用nano-DESI代替液体萃取表面分析(LESA),与Orbitrap Eclipse三合一高分辨率质谱仪联用,对组织切片进行非变性蛋白质MSI。LESA法利用毛细管吸取样品表面的静态液滴,随之将其放置在质谱仪入口处,以进行nano-ESI质谱分析。LESA MSI已被用于非变性蛋白质的成像中,可获得1 mm的空间分辨率和较高的灵敏度。基于nano-DESI,该团队实现了200 μm的空间分辨率,显著优于LESA MSI方法。在nano-DESI MSI方法中,他们使用含有表面活性剂(detergent)的乙酸铵水溶液作为溶剂,该溶剂能有效地从组织中萃取蛋白质及其复合物,并在质谱分析中保持它们的天然结构。他们利用nano-DESI直接对大鼠肾脏组织中的天然蛋白质进行了自上而下的蛋白质组学(top-down proteomics)分析,鉴定了S100-A6的非共价二聚体和PE结合蛋白等多种新型复合物,并首次展示了这些复合物的空间分布。这项技术还揭示了一系列配体结合蛋白和金属结合蛋白在大鼠脑组织中的空间分布,包括GDP结合蛋白质(ARF3、ARF1和GTP酶Ran)、小清蛋白*α*+2Ca^2+^和碳酸酐酶+Zn^2+^等^[15]^。此外,该团队^[16]^将nano-DESI方法用于低丰度膜蛋白复合物的鉴定和成像,在羊眼晶状体组织中发现了完整的四聚体膜蛋白质Aqp0(组装质量113.0 kDa)和*β*-B2-晶体蛋白复合体(组装质量94.5 kDa),并指出两种膜蛋白具有截然相反的空间分布:Aqp0在皮质组织中含量最高,而*β*-B2-晶体蛋白组装在晶状体核中含量最高。

为了突破nano-DESI质谱分析的质量上限,Cooper团队^[17]^进一步优化了质谱仪中的离子光学器件和气体压力,以改善高质荷比离子的传输效率,并采用不同类型的离子裂解方式,以提高蛋白质序列覆盖度,从而实现了对超高质量(高达145 kDa)蛋白组装体的分析。从成年雄性大鼠的不同类型的组织(脑、肾、肝)中,在宽泛的质量范围(37.0~145.4 kDa)内表征了具有不同化学计量(二聚体、三聚体和四聚体)的一系列完整的蛋白组装体。他们还发现在MSI实验之前,使用乙酸铵溶液洗涤组织不仅不会破坏膜蛋白质的空间分布,还能显著减少可溶性蛋白质的信号,从而提高了膜蛋白质的检测灵敏度^[18]^。该团队^[19]^利用高通量模块MetaUniDec对原生MSI数据集的每个像素进行去卷积,并将*m/z*域图像文件转换到质量域,从而实现了复杂蛋白质谱图的快速去卷积。这个流程被用于高通量分析新生成的小鼠大脑成像数据集和已发表的羊眼晶状体成像数据集,实现了数据的自动化分析,提高了蛋白质MSI数据的处理效率。

除了分析蛋白质复合物外,蛋白质变构体(proteoform)研究是nano-DESI MSI的另一个主要应用领域。由于基因变异、RNA选择性剪接和翻译后修饰(PTM)等因素,单个基因能产生一系列结构类似的蛋白质变构体,这给蛋白质组学研究带来了新挑战。Laskin团队^[20,21]^将nano-DESI MSI与自上而下蛋白质组学相结合,以大鼠脑组织作为模型系统,首次实现了19种不同蛋白质的40种变构体的鉴定与成像,并指出单个蛋白质变构体在不同的脑部位存在显著差异。通过优化毛细管探针的设计和组织切片的厚度(12~25 μm),提高了空间分辨率(约15 μm)和灵敏度,并且证实密集采样(oversampling)在不降低灵敏度的前提下进一步提高了空间分辨率(约7 μm)。他们还使用nano-DESI MSI研究生物组织中蛋白质*N*-糖基化的空间分布规律,展示了福尔马林固定的石蜡包埋的人肝细胞癌和人前列腺组织中38种N-连接聚糖的离子影像,并通过MS/MS解析这些聚糖的序列。该研究展示了nano-DESI MSI在空间上表征生物组织中N-连接聚糖的潜力^[22]^。

Kelleher团队^[23]^成功整合nano-DESI和单个离子检测质谱(individual ion MS),开发了一种高通量、无标记的蛋白质变构体质谱成像技术(proteoform imaging MS, PiMS),将鉴定的蛋白质相对分子质量上限提高了4倍。PiMS方法从人类肾脏组织中同时鉴定及成像169个蛋白质异构体(分子质量≤70 kDa),并展示了它们在肾脏血管、髓质和皮质区域中截然不同的空间分布。他们进一步建立了一个自动化的PiMS流程(AutoPiMS),在PiMS基础上增加了一个计算引擎,自动化选择目标蛋白质,并生成相应的采集方法,随后与高通量数据处理和数据库搜索相连接,可直接从组织微环境中快速鉴定蛋白质变构体的分子特征。将其应用于人类卵巢癌切片,实现了73个蛋白质变构体(≤54 kDa)的快速鉴定,每个蛋白质分析时间不超过1 min^[24]^。该团队^[25]^还提出了单细胞PiMS技术(scPiMS),对单个细胞的蛋白质组进行直接采样和深度分析;该技术将细胞处理速率提高了数倍(每个细胞分析时间< 10 s),并获得了高覆盖率的蛋白质组成数据,为解决单细胞蛋白质组学的通量问题提供了一种有效方法。

## 4 结论与展望

综上,nano-DESI是一种极具发展潜力的质谱成像技术,具有样品处理简单、高空间分辨率、高灵敏度和仪器兼容性好等优点。Nano-DESI已实现与不同类型质谱仪的联用,包括QqQ、QToF、Orbitrap和FTICR等,这些联用极大地拓展了nano-DESI的应用范围。Nano-DESI MSI不仅用于深度分析脂质精细结构,还揭示了配体结合蛋白质的天然结构以及蛋白质变构体的空间分布规律。目前,该领域中一个亟需解决的问题是如何加速推动nano-DESI成像装置的成果转化。随着离子源设计趋于成熟、集成式探针的发明以及市场需求的增加,nano-DESI装置的商业化指日可待。如何将nano-DESI与化学衍生化反应联用,以拓展目标分析物的维度;如何开发高效的数据分析方法,从纷繁复杂的数据中快速筛选出关键信息;nano-DESI空间多组学研究如何满足环境与健康的需求,这些都是nano-DESI领域中值得关注的问题。
